# Metabolic Model-Based Integration of Microbiome Taxonomic and Metabolomic Profiles Elucidates Mechanistic Links between Ecological and Metabolic Variation

**DOI:** 10.1128/mSystems.00013-15

**Published:** 2016-01-19

**Authors:** Cecilia Noecker, Alexander Eng, Sujatha Srinivasan, Casey M. Theriot, Vincent B. Young, Janet K. Jansson, David N. Fredricks, Elhanan Borenstein

**Affiliations:** a Department of Genome Sciences, University of Washington, Seattle, Washington, USA; b Vaccine and Infectious Disease Division, Fred Hutchinson Cancer Research Center, Seattle, Washington, USA; c Department of Population Health and Pathobiology, North Carolina State University, Raleigh, North Carolina, USA; d Department of Internal Medicine, Division of Infectious Diseases, University of Michigan, Ann Arbor, Michigan, USA; e Department of Microbiology and Immunology, University of Michigan, Ann Arbor, Michigan, USA; f Biological Sciences Division, Pacific Northwest National Laboratory, Richland, Washington, USA; g Division of Allergy and Infectious Diseases, University of Washington, Seattle, Washington, USA; h Department of Microbiology, University of Washington, Seattle, Washington, USA; i Department of Computer Science and Engineering, University of Washington, Seattle, Washington, USA; j Santa Fe Institute, Santa Fe, New Mexico, USA; University of Illinois at Chicago

**Keywords:** microbiome, multi-omic, metabolic modeling, community composition, metabolomics

## Abstract

Multiple molecular assays now enable high-throughput profiling of the ecology, metabolic capacity, and activity of the human microbiome. However, to date, analyses of such multi-omic data typically focus on statistical associations, often ignoring extensive prior knowledge of the mechanisms linking these various facets of the microbiome. Here, we introduce a comprehensive framework to systematically link variation in metabolomic data with community composition by utilizing taxonomic, genomic, and metabolic information. Specifically, we integrate available and inferred genomic data, metabolic network modeling, and a method for predicting community-wide metabolite turnover to estimate the biosynthetic and degradation potential of a given community. Our framework then compares variation in predicted metabolic potential with variation in measured metabolites’ abundances to evaluate whether community composition can explain observed shifts in the community metabolome, and to identify key taxa and genes contributing to the shifts. Focusing on two independent vaginal microbiome data sets, each pairing 16S community profiling with large-scale metabolomics, we demonstrate that our framework successfully recapitulates observed variation in 37% of metabolites. Well-predicted metabolite variation tends to result from disease-associated metabolism. We further identify several disease-enriched species that contribute significantly to these predictions. Interestingly, our analysis also detects metabolites for which the predicted variation negatively correlates with the measured variation, suggesting environmental control points of community metabolism. Applying this framework to gut microbiome data sets reveals similar trends, including prediction of bile acid metabolite shifts. This framework is an important first step toward a system-level multi-omic integration and an improved mechanistic understanding of the microbiome activity and dynamics in health and disease.

**IMPORTANCE** Studies characterizing both the taxonomic composition and metabolic profile of various microbial communities are becoming increasingly common, yet new computational methods are needed to integrate and interpret these data in terms of known biological mechanisms. Here, we introduce an analytical framework to link species composition and metabolite measurements, using a simple model to predict the effects of community ecology on metabolite concentrations and evaluating whether these predictions agree with measured metabolomic profiles. We find that a surprisingly large proportion of metabolite variation in the vaginal microbiome can be predicted based on species composition (including dramatic shifts associated with disease), identify putative mechanisms underlying these predictions, and evaluate the roles of individual bacterial species and genes. Analysis of gut microbiome data using this framework recovers similar community metabolic trends. This framework lays the foundation for model-based multi-omic integrative studies, ultimately improving our understanding of microbial community metabolism.

## Abstract (precis)

Studies characterizing both the taxonomic composition and metabolic profile of various microbial communities are becoming increasingly common, yet new computational methods are needed to integrate and interpret these data in terms of known biological mechanisms. Here, we introduce an analytical framework to link species composition and metabolite measurements, using a simple model to predict the effects of community ecology on metabolite concentrations and evaluating whether these predictions agree with measured metabolomic profiles. We find that a surprisingly large proportion of metabolite variation in the vaginal microbiome can be predicted based on species composition (including dramatic shifts associated with disease), identify putative mechanisms underlying these predictions, and evaluate the roles of individual bacterial species and genes. Analysis of gut microbiome data using this framework recovers similar community metabolic trends. This framework lays the foundation for model-based multi-omic integrative studies, ultimately improving our understanding of microbial community metabolism.

## INTRODUCTION

The human microbiome carries out a plethora of metabolic processes that are often vital to the health of the host. Microbiome metabolic activity can, for example, impact energy harvest, inflammation, and infection susceptibility (1–3), suggesting that alterations in community metabolism may be an important mechanism underlying an array of poorly understood associations between the composition of the microbiome and disease (4–6). Indeed, the metabolic capacity of the gut microbiome appears to be relatively constant across healthy individuals (7), and yet, it can vary dramatically in response to perturbations like antibiotic treatment or diet changes (8, 9) or in a variety of disease states (10, 11).

Understanding the relationship between the composition of the microbiome and its metabolic activity (and ultimately, the development of microbiome-associated diseases) is therefore an important task. To this end, numerous recent studies have paired comprehensive taxonomic characterization (based on, for example, 16S rRNA gene assays) with metabolomic profiling, aiming to reveal and evaluate the mechanisms underlying taxonomic and metabolic shifts in the microbiome across diverse environments and disease states (12–27). To date, however, methods for integrating taxonomic and metabolomic data are lacking, and consequently, the vast majority of these studies have analyzed community composition and metabolite profiles independently or focused on identifying statistical associations between these two data types.

While the discovery of such associations is clearly an important first step in describing the function and dynamics of the microbiome in health and disease, it ignores extensive prior knowledge of genomic capacities and metabolic mechanisms that link community ecology and metabolism and may accordingly fall short of gaining a systems-level mechanistic understanding of such complex ecosystems. For example, a strong correlation between a species and a metabolite may have very different interpretations depending on whether the species in question is known to degrade that metabolite or to synthesize it. Integrating the taxonomic and metabolomic profiles of the system under study therefore requires not only linking these two data sets but also the incorporation of prior reference information about the metabolic capacities of various community members and the way such capacities interact. Specifically, an integrated analysis could shed light on the extent to which variation in a metabolite of interest can be explained by observed shifts in community ecology and metabolic capacity, as opposed to alternative environmental factors. This is crucial for gaining a comprehensive understanding of the microbiome and for future efforts to modulate metabolic phenotypes via microbiome-based interventions.

Several recent studies have taken initial steps to address this challenge. One avenue of research aims to reconstruct predictive metabolic models of community metabolism in various settings (using, for example, constraint-based modeling), which can then potentially be validated by metabolomic profiling (28–30). This approach, however, depends on relatively complete and high-quality metabolic models of the species involved and, therefore, may not scale well to complex communities with partially characterized taxa. Other studies have used information about enzymatic reactions to infer metabolic turnover potential from taxonomic composition and metagenome content (31, 32). However, these studies focused on comparing predicted metabolic potential to environmental parameters or community dysbiosis rather than to detailed, large-scale metabolomic phenotypes. McHardy et al. (33) used correlation network analysis to cluster metabolites and evaluated the correspondence between the resulting clusters and metabolically related pathway abundances, an approach that successfully quantified relationships between functional pathways and metabolites but that was still primarily association based and difficult to interpret. Sridharan et al. (34) similarly focused on a small subset of metabolism, constructing a reference genome-based supraorganism metabolic network model and applying a pathway construction algorithm to predict bioactive aromatic microbial metabolites likely to be found in the human gut. These studies all show the tremendous promise of linking microbial composition to metabolomic variation based on prior knowledge of the various metabolic processes, and yet, they are still limited in scale. Thus, the development of a systematic, mechanistic approach for evaluating the relationships between the community ecology and metabolite shifts is called for.

We therefore present here a comprehensive analytical multi-omic framework for integrating community structure and metabolic profile, aiming to elucidate mechanisms underlying metabolic variation in the human microbiome. Our framework first infers community gene content based on available and inferred genomic information and adapts a method originally developed to interpret environmental metagenomes (31) to approximate the potential effect of the microbiome on each metabolite. We systematically compare these estimates to measured metabolome variation and interpret the results in terms of metabolic mechanisms based on taxonomic shifts. We apply this framework to two data sets pairing community taxonomic composition and global metabolite profiles from the vaginal microbiome, as well as to data sets from the gut microbiomes of humans and mice. Using this framework, we identify a large number of metabolites whose variation across samples can be explained (or “predicted”) by shifts in microbial community composition and the metabolic capacity of the various member species. We further use this approach to identify species and reactions that are key contributors to the calculated communitywide metabolic potential and highlight putative alternative mechanisms for poorly predicted metabolites. Importantly, our analysis detects broad trends in metabolite predictability across data sets and serves as a proof of concept of the use of systematic mechanism-based integration of multi-omic data to gain new insight into microbial community metabolism.

## RESULTS

### A metabolic model-based framework for integrating taxonomic and metabolomic data.

We developed a computational framework to systematically link variation in community ecology with observed variation in its metabolic phenotype (Fig. 1). Our framework specifically assesses whether the measured between-sample variation in metabolite abundances can be explained by observed shifts in species composition and information about the metabolic capacity of each species.

**FIG 1:**
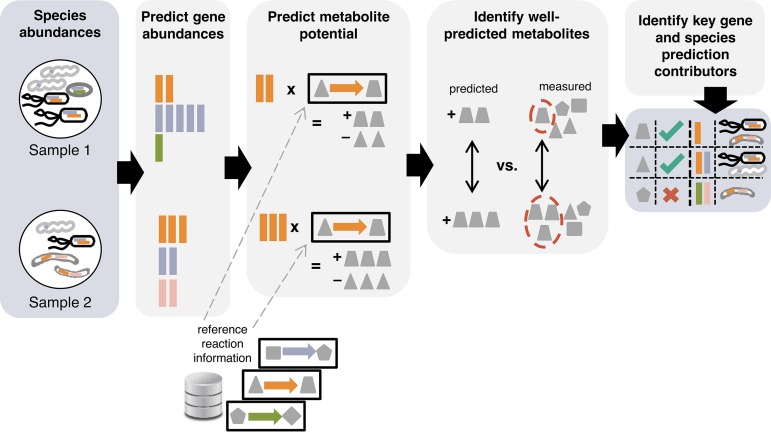
Framework for integrating taxonomic and metabolomic data. Species composition is first used to predict the metagenome’s gene content, which is then paired with reaction information to estimate the community metabolic potential (CMP) for each sample and metabolite. Variation in predicted CMP scores is compared to variation in measured metabolite abundances (using pairwise differences) to identify well-predicted metabolites. A perturbation-based approach is used to additionally identify key species, gene, and reaction contributors to CMP scores.

Briefly, our framework first infers the metagenome content for each sample based on taxonomic composition and available or inferred reference genome information (35). Inferred metagenomes are then normalized using a previously introduced method (MUSiCC) (36), resulting in an estimate of the average copy number of each gene across microbiome genomes. Next, our framework applies a method for predicting relative metabolic turnover (31), using a metabolic network model to translate the resulting enzymatic gene abundance estimates into community-based metabolite potential (CMP) scores. These scores represent the relative capacity of the community in a given sample to generate or deplete each metabolite, based on metabolic reference information that links enzymes to their substrates and products (37). To evaluate these scores, our framework then compares for each metabolite the differences in CMP scores between all pairs of samples with the differences in the corresponding measured metabolite abundance. Using these pairwise comparisons and a statistical test for correlation between two distance matrices, our framework evaluates whether there is an agreement between variation in predicted CMP scores and variation in measured metabolite abundances. We term those metabolites for which this agreement is statistically significant “well-predicted.” Finally, our framework uses a perturbation-based approach to identify the bacterial species, genes, and reactions that are the key mechanistic contributors to calculated CMP scores. A more detailed description of this framework can be found in Materials and Methods.

### Metabolic model-based prediction explains metabolite variation in the vaginal microbiome based on taxonomic shifts.

We first applied our framework to data sets pairing bacterial community and metabolomic profiles from the vaginal microbiome, a relatively simple community typically dominated by a limited number of species. We specifically analyzed two independently obtained data sets (each consisting of ~70 samples; see Table S1 in the supplemental material), characterizing the vaginal microbiomes and metabolomes of healthy women and women with bacterial vaginosis (BV) (22). Samples from the first data set (data set 1) were analyzed for taxonomic composition using quantitative PCR (qPCR) for 14 vaginal bacterial species and for metabolites using global liquid chromatography-mass spectrometry (LC-MS) and gas chromatography (GC)-MS, whereas samples from the second data set (data set 2) were analyzed using broad-range 16S rRNA gene sequencing and targeted LC-MS (see Materials and Methods).

10.1128/mSystems.00013-15.8 Table S1 Study design and metabolomic data description for all analyzed data sets. Download Table S1, PDF file, 0.4 MB. Copyright © 2016 Noecker et al. 2016 Noecker et al. This content is distributed under the terms of the Creative Commons Attribution 4.0 International license.

In each of these data sets, we used our framework to calculate the CMP score for each metabolite and in each sample. Of the metabolites assayed in each data set, roughly 50% could not be associated with a CMP score due to missing or noninformative annotated metabolic data (see Materials and Methods; see also Table S1 in the supplemental material) and were accordingly discarded from downstream analysis. The CMP scores of the remaining metabolites were compared to measured metabolite abundances as described above to examine whether the observed variation in the metabolite abundances across samples can be explained mechanistically by variation in the set of species comprising the community. Surprisingly, we found that 40.2% of the metabolites analyzed in data set 1 and 34.5% of metabolites analyzed in data set 2 were well-predicted (see Table S2), suggesting that for a substantial fraction of metabolites, information about the metabolic capabilities for the member species is sufficient to explain observed differences in metabolite abundance. We further confirmed that the identification of well-predicted metabolites and the correlations observed between calculated CMP scores and measured abundances are not artifacts of the data covariance structure, using randomized metabolic networks to generate a predictability null model (see Materials and Methods). We found that randomized networks produced a consistently lower proportion of well-predicted metabolites than the real network (*P* < 0.01 for both data sets). Metabolites analyzed in both data sets were generally predictable at similar levels (ρ = 0.63, Spearman correlation test) (see Fig. S1). Finally, we also observed a significant overlap between metabolites for which variation in CMP scores was significantly correlated with variation in measured metabolite abundance in both data sets 1 and 2 and in a simple monoculture-based *Escherichia coli* data set (*P* = 0.04; Fisher exact text) (see Text S1 and Fig. S2). This finding suggests that our framework may identify consistent control points in microbial metabolism.

10.1128/mSystems.00013-15.1 Figure S1  Comparison of metabolite predictions between vaginal data sets 1 and 2. Metabolites assayed and analyzed in both data sets are plotted, with the *x* axis representing the prediction level of the metabolite in data set 1 (measured as the Spearman correlation between pairwise differences in calculated CMP scores and pairwise differences in measured metabolite abundances) and the *y* axis representing the prediction level of that metabolite in data set 2. The color of each point denotes the agreement between the prediction type (well-predicted, anti-predicted, or neither) across the two data sets. Download Figure S1, TIFF file, 0.5 MB. Copyright © 2016 Noecker et al. 2016 Noecker et al. This content is distributed under the terms of the Creative Commons Attribution 4.0 International license.

10.1128/mSystems.00013-15.2 Figure S2  Metabolite predictability across metabolic categories using *E. coli* monoculture gene expression data. (A) Prediction outcomes for metabolites assayed and analyzed in *E. coli* cultures treated to cause assorted perturbations. Bars show the number of metabolites of each prediction type in each KEGG category (see Fig. 2 for comparison). (B to G) Visualization of key species and reaction contributors for metabolites of interest. The size of each node represents the magnitude of the correlation between variation in CMP scores and variation in measured metabolite abundances. Edges denote key reaction contributors. Download Figure S2, TIFF file, 1.2 MB. Copyright © 2016 Noecker et al. 2016 Noecker et al. This content is distributed under the terms of the Creative Commons Attribution 4.0 International license.

10.1128/mSystems.00013-15.9 Table S2 A list of all metabolites assayed and analyzed in all data sets, detailing prediction levels, host state associations, and key gene and species contributors. Download Table S2, XLSX file, 0.1 MB. Copyright © 2016 Noecker et al. 2016 Noecker et al. This content is distributed under the terms of the Creative Commons Attribution 4.0 International license.

10.1128/mSystems.00013-15.10 Text S1  Predicting variation in metabolite abundances in a simple monoculture system identifies known mechanisms regulating metabolite variation. Download Text S1, DOCX file, 0.1 MB. Copyright © 2016 Noecker et al. 2016 Noecker et al. This content is distributed under the terms of the Creative Commons Attribution 4.0 International license.

We next examined whether well-predicted metabolites tend to be associated with specific metabolic categories or host state. We found that well-predicted metabolites spanned a range of metabolic categories (Fig. 2A). Specifically, well-predicted metabolites represent all major metabolic categories, with many well-predicted metabolites being associated with amino acid metabolism, an important category of microbe-mediated processes in this environment. Additionally, 60% and 40% of the strongly BV-enriched metabolites, including known metabolic markers of BV (38), such as the amino acid catabolites *N*-acetylputrescine, spermidine, and citrulline, were predicted well in each data set (Fig. 2B).

**FIG 2:**
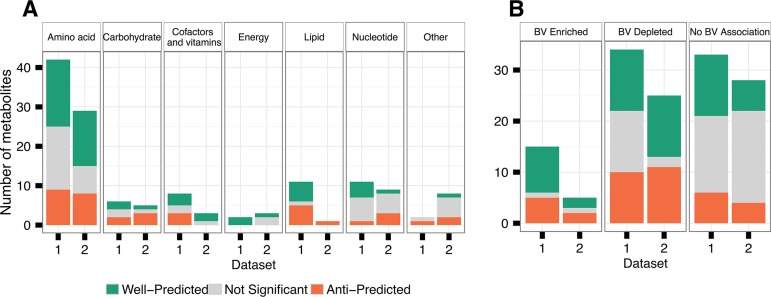
Metabolite predictability across metabolic categories (A) and disease states (B) in the vaginal microbiome. Well-predicted metabolites are defined as those for which variation in CMP scores is significantly correlated (using a Mantel test) with variation in measured metabolite abundance at a false discovery rate (FDR) of 0.01. Anti-predicted metabolites are similarly defined as those for which variation in CMP scores is significantly negatively correlated with variation in measured metabolite abundances (FDR 0.01). Metabolic categorization is based on KEGG data, and disease enrichment is based on a Wilcoxon rank sum test for association with bacterial vaginosis (BV) with a Bonferroni-corrected *P* value of <0.1.

Interestingly, we also observed a substantial portion of metabolites for which variation in the CMP scores was strongly negatively correlated with variation in measured abundances (25.6% in data set 1 and 29.3% in data set 2; see Table S2 in the supplemental material). These “anti-predicted” metabolites were often linked to a well-predicted metabolite either by a reversible reaction (which is not factored into CMP score calculation) (7 and 4 metabolite pairs in data set 1 and 2, respectively) or by a reaction synthesizing the anti-predicted metabolite from a well-predicted metabolite (6 and 2 metabolite pairs). For example, in data set 1, glutamate is well-predicted, while glutamine, a metabolite that can be synthesized from glutamate, is anti-predicted, suggesting that other, unaccounted-for factors influence its abundance in this environment. Overall, anti-predicted metabolites were adjacent to well-predicted metabolites more frequently than expected by chance (15 and 8 metabolite pairs, *P* < 0.005 and *P* < 0.03 in data set 1 and 2, respectively, by a permutation-based test; see Materials and Methods). Such anti-predicted metabolites may be the result of missing information about community composition or genomic capacities. However, they may also point to environmentally regulated points in metabolism (as opposed to microbiome-controlled metabolites), where an environmental change in metabolite abundance and nutrient availability give rise to taxonomic shifts in the microbiome. Put differently, in contrast to well-predicted metabolites that are likely produced by the microbiome, so that an increase in their abundance correlates with an increase in the abundance of species that have the capacity to synthesize them, an increase in the abundance of anti-predicted metabolites can potentially be introduced by the environment and selects for species that have the capacity to degrade them (see Discussion).

### A small set of BV-enriched bacterial species explains a large portion of the metabolome variation.

We next examined the contribution of individual species to the calculated CMP scores of each metabolite. We quantified each species’ contribution as the correlation between a CMP score that is calculated based on that species alone (e.g., ignoring all other species in the community) and the community-wide CMP score described above (Materials and Methods). We defined species for which this correlation was above 0.5 as key contributors. We first focused on data set 1, in which only a small number of species was assayed but the availability of absolute concentration data (owing to the use of qPCR) may better distinguish key species in the community. In total, we found that 10 of the 11 species analyzed in data set 1 were key contributors to at least one metabolite. Importantly, the vast majority of metabolites (93.9%) had 4 or fewer key contributors, yet the particular combination of species varied widely across metabolites. This suggests that shifts in the abundance of each metabolite (and in particular shifts associated with the BV state) may be attributed to a small number of species rather than to community-wide dysbiosis. For instance, although both *N*-acetylputrescine and citrulline are BV-enriched polyamine metabolites, the increased abundance of *N*-acetylputrescine in BV is driven in both data sets mostly by the genomic capacities and variation in the abundance of *Prevotella* species, while citrulline’s enrichment is driven primarily by *Atopobium vaginae* and *Eggerthella*. Species contributing to the CMP scores of anti-predicted metabolites also recover known processes: for example, *Lactobacillus iners* is the only key species contributor driving the anti-prediction of glycerol in data set 1 (due to *L. iners*’ genome encoding glycerol utilization genes). A recent metatranscriptomic study of vaginal *L. iners* found evidence that this species is largely the only member of this community that uses glycerol as a carbon source (39), which combined with our results, suggests that a vaginal environment with glycerol availability may promote *L. iners* growth.

We further examined the number of metabolites (and specifically, well-predicted metabolites) for which each species was a key contributor to CMP score calculation. We found that in data set 1, *Eggerthella* sp. 1 and *Megasphaera* type 1 were key contributors to a particularly high number of metabolites relative to the contributions of other species (Fig. 3). BV-enriched metabolites that were well-predicted primarily by these two species alone include *N*-acetylneuraminate, ethanolamine, and the lipid metabolites 4-trimethylaminobutanoate/gamma-butyrobetaine and 3-methyl-2-oxobutanoate (see Fig. S3 in the supplemental material). Notably, these are neither the most abundant nor the most variable species in this data set, although *Eggerthella* sp. 1 is the most differentially abundant species between healthy and BV samples based on Wilcoxon rank-sum tests (*P* < 10^−8^), whereas *Megasphaera* is fifth most differentially abundant (*P* < 10^−9^). *Eggerthella* also has the largest genome of any of the analyzed species in terms of the number of protein-coding genes (2,936 genes). Combined, these findings illustrate that the species contributing most significantly to potential shifts in disease-associated metabolic phenotypes may not necessarily be the most abundant or most variable species and that observed metabolic shifts are the product of complex dependencies between ecological dynamics and metabolic capacity.

10.1128/mSystems.00013-15.3 Figure S3  Examples of well-predicted metabolites in data set 1 and their key species and gene contributors. The size of each node represents the magnitude of the correlation between variation in CMP scores and variation in measured metabolite abundances for a given metabolite, while the color indicates whether this correlation is positive or negative (i.e., well-predicted or anti-predicted). Edges denote key reaction contributors. Colored rectangular bars denote the key species contributors for the given metabolite. Download Figure S3, TIFF file, 1.0 MB. Copyright © 2016 Noecker et al. 2016 Noecker et al. This content is distributed under the terms of the Creative Commons Attribution 4.0 International license.

**FIG 3:**
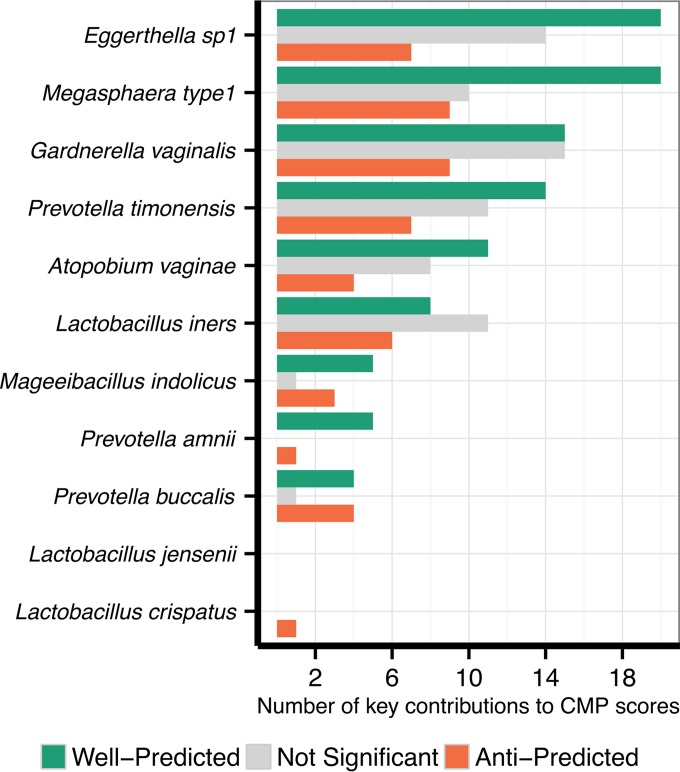
Key species contributors to metabolites in the vaginal microbiome. Each species that participated in the calculation of CMP scores in data set 1 is shown along the *y* axis. The *x* axis indicates the numbers of well-predicted and anti-predicted metabolites (as well as those with nonsignificant predictions) for which that species was a key contributor (see Materials and Methods).

These trends are partially recapitulated in data set 2 (see Fig. S4 in the supplemental material). Specifically, 31 of the 171 operational taxonomic units (OTUs) in this data set were key contributors to at least one metabolite. Again, most metabolites (64%) had 4 or fewer key contributors, but the combination of OTUs varied across metabolites. Of the 42 metabolites analyzed in both data sets, 26 share at least one key contributing genus, including 9 of the 11 metabolites that were well-predicted in both data sets (see Fig. S4B). Interestingly, however, the OTUs that contributed to the CMP scores of the most well-predicted metabolites in data set 2 included an OTU (227000) identified as BVAB1, an OTU (4377809) corresponding to the bacterium *Mageeibacillus indolicus* (previously known as BVAB3), an OTU corresponding to *Prevotella amnii* (663885), another *Prevotella* OTU (403822), and an OTU in the genus *Parvimonas* (132546) (of which only *M. indolicus* and *P. amnii* were analyzed in data set 1). An OTU corresponding to the *Eggerthella* species noted in data set 1 was also a key contributor to many well-predicted metabolites. Relatively low contributions to CMP scores by *Lactobacillus crispatus* (typically associated with health) and *Atopobium vaginae* were consistent between the two data sets. Given the difference in taxonomic profiling methods between the two data sets (qPCR versus 16S rRNA gene sequencing), the difference in the way genomic content was inferred (reference genomes versus PICRUSt-based predictions), missing reference genomic information for three species assayed in data set 1, and the focus on selected metabolites of interest in data set 2, the variation in the key contributors obtained is perhaps not surprising. For example, the increased importance of *M. indolicus* in data set 2 could be a function of differences in the features of metabolites assayed and analyzed between the two data sets, and/or it could be from differences in reference information; a total of 88 of 732 Kyoto Encyclopedia of Genes and Genomes (KEGG) orthology groups (KOs) differed in copy number between the reference genome used for prediction in data set 1 and the predicted genome content for the corresponding OTU in data set 2. The full list of key species contributors can be found in Table S2.

10.1128/mSystems.00013-15.4 Figure S4  Key species contributors to metabolites in data set 2 and variation in species contribution across data sets 1 and 2. (A) Each OTU that participated in the calculation of CMP scores in data set 2 is shown along the *y* axis. The *x* axis indicates the numbers of well-predicted and anti-predicted metabolites (as well as those with nonsignificant prediction) for which that OTU was a key contributor. OTUs matched to species assayed in data set 1 with conflicting taxonomy (Materials and Methods) are shown in parentheses. Compare also with Fig. 3. (B) A comparison between key species contributors (at the genus level) of each metabolite across data sets 1 and 2. The two left columns indicate whether the metabolite was well-predicted, anti-predicted, or neither in each of the two data sets. A bright blue tile indicates that at least one species or OTU in a given genus was identified as a key contributor to predictions for the metabolite in question. Note that several genera whose abundances were measured in data set 2 were either not assayed in data set 1 (in which taxonomic composition was assayed using 16S rRNA gene qPCR) or did not have genomic information available at the time. Download Figure S4, TIFF file, 4.9 MB. Copyright © 2016 Noecker et al. 2016 Noecker et al. This content is distributed under the terms of the Creative Commons Attribution 4.0 International license.

### Well-predicted metabolites tend to be involved in condition-specific metabolism.

We next set out to identify key gene contributors to each metabolite’s CMP score, by calculating the correlation between the original CMP scores and a CMP score calculated when the link between the gene in question and the metabolite was deleted from the metabolic model (Materials and Methods). Genes for which this correlation was <0.5 were considered key contributors for that metabolite, and any reaction catalyzed by the enzyme encoded by that gene was considered a key reaction contributor. This analysis relates specific combinations of reaction information and genomic shifts to the predicted potential for metabolite variation, allowing us to examine whether our approach recovers known metabolic mechanisms (see Fig. S3 in the supplemental material). For example, the CMP scores for well-predicted amino acid derivatives, including *N*-acetylputrescine and citrulline, were driven by synthesis enzymes forming part of amino acid catabolism pathways and encoded by BV-associated bacteria (see Fig. S3A and B). A subset of amino acids, including glutamate and phenylalanine, were well-predicted on the basis of a combination of available biosynthesis pathways and the predicted abundance of tRNA synthetase genes and degradation pathways. Pyruvate levels were slightly lower in BV samples and well-predicted primarily by acetolactate synthase, which catalyzes the first step diverting pyruvate to branched-chain amino acid synthesis. This mechanism is consistent with the overall shift from carbohydrate-based to amino acid-based metabolism that is typical of the BV state. In another example, Srinivasan et al. (22) have noted that the depletion of reduced glutathione in BV samples is surprising, as the BV vagina is a relatively reduced environment (40). Our framework predicts this shift in glutathione well in both data sets (prediction levels of 0.49 and 0.30 in data sets 1 and 2, respectively) and attributes it to a lack of glutathione peroxidase genes in *Lactobacillus* species that predominate in healthy vaginal samples (see Fig. S3C). Genes in cofactor synthesis pathways also tended to contribute to predictive CMP scores for metabolites in these pathways, including nicotinate, NAD^+^, and FAD^+^.

We further characterized the set of key gene contributors of each metabolite and explored their relationships with metabolite predictability (see Fig. S5 in the supplemental material). Most metabolites had only a small number of genes with the potential to enzymatically impact them, and of these, most were identified as key contributors. Interestingly, well-predicted metabolites tended to have a higher proportion of the set of relevant genes as key contributors in both data sets (*P* = 0.002 and *P* = 0.09 in data sets 1 and 2, respectively; Wilcoxon rank-sum test). Surprisingly, the key genes for well-predicted metabolites were less variable across samples than those for other metabolites (*P* = 0.08 and *P* = 0.002 in data sets 1 and 2, respectively; Wilcoxon rank-sum test). We also examined whether the key gene contributors for each metabolite encoded enzymes solely catalyzing reactions synthesizing the metabolite in question, degrading it, or both (see Materials and Methods). We found that BV-enriched metabolites with key gene contributors that are associated only with synthesis enzymes were almost always well-predicted (11 of 13 metabolites across both data sets) (Fig. 4; see also Fig. S6). Conversely, metabolites depleted in the BV state and with key gene contributors encoding only degradation enzymes also tended to be well-predicted (18 of 31 metabolites across both data sets) (Fig. 4; see also Fig. S6). These trends suggest that the most predictable variation resulted from the transition between these two conditions, in particular, the impact of the presence or absence of novel metabolic synthesis and degradation capacities in BV, rather than shifts in the abundance of more widely found metabolic pathways.

10.1128/mSystems.00013-15.5 Figure S5  Key gene contributors of metabolites in data sets 1 (A) and 2 (B). Each metabolite analyzed in each data set is listed, along with the number of genes contributing to its CMP scores (*x* axis), divided into the numbers of key genes coding for synthesis enzymes, key genes coding for degradation enzymes, and nonkey genes in the same two categories. The left columns indicate whether each metabolite was enriched or depleted in BV samples and whether it was well-predicted, anti-predicted, or neither. Metabolites are ordered by the total number of relevant genes and their association with BV. Download Figure S5, TIFF file, 4.0 MB. Copyright © 2016 Noecker et al. 2016 Noecker et al. This content is distributed under the terms of the Creative Commons Attribution 4.0 International license.

10.1128/mSystems.00013-15.6 Figure S6  Trends in metabolite predictability in data set 2. Area plots depict the numbers of metabolites whose CMP scores are driven by synthesis, by degradation, or by both in relation to their association with the host state and their predictability. The visualization used is similar to that shown in Fig. 4. Download Figure S6, TIFF file, 0.8 MB. Copyright © 2016 Noecker et al. 2016 Noecker et al. This content is distributed under the terms of the Creative Commons Attribution 4.0 International license.

**FIG 4:**
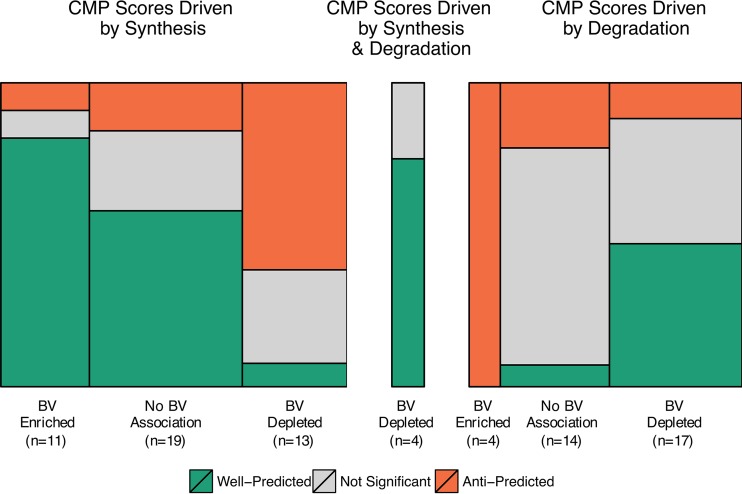
Trends in metabolite predictability in terms of key gene contributors. Area plots depict the numbers of metabolites in data set 1 whose CMP scores are driven by synthesis, by degradation, or by both in relation to their association with the host state and their predictability. The width of each box corresponds to the number of metabolites associated with each host disease state (enriched in BV samples, depleted in BV samples, or neither), and the height corresponds to the number of metabolites that are well-predicted, anti-predicted, or not significantly predicted (also indicated by color). See Fig. S6 in the supplemental material for a similar plot describing metabolite prediction in data set 2.

### Application to gut microbiome communities reiterates metabolic trends and highlights community complexity.

Finally, we explored the application of this framework to samples from gut microbial communities, bearing in mind the caveats of increased environmental influences resulting from diet, as well as increased community complexity. Specifically, we applied this framework to two additional data sets, one evaluating the impact of antibiotic treatment with cefoperazone on the cecal contents of specific-pathogen-free mice (data set 3) (23) and another profiling the microbiome and metabolome of humans with inflammatory bowel disease and healthy controls (data set 4) (15, 41). Because the second study used shotgun metagenomic sequencing, in its analysis, we did not need to predict metagenome content from community composition and instead estimated gene abundances directly (see Materials and Methods) (42). As expected given the more complex and unstable community environment, we observed lower proportions of well-predicted metabolites in these data sets (see Fig. S7 in the supplemental material), but we also identified interesting patterns in the relationships between variation in community composition and metabolism in these settings.

10.1128/mSystems.00013-15.7 Figure S7  Metabolite predictability across metabolic categories in the gut microbiome. The visualization used is similar to that shown in Fig. 2, with the left bar corresponding to predictions in data set 3 and the right bar corresponding to data set 4. Download Figure S7, TIFF file, 0.8 MB. Copyright © 2016 Noecker et al. 2016 Noecker et al. This content is distributed under the terms of the Creative Commons Attribution 4.0 International license.

Data set 3 recapitulated many metabolite predictability trends observed in our analysis of the vaginal microbiota, including successful prediction of metabolite variation and the effects of perturbation on community ecology and metabolism. In total, 39 of the 116 metabolites (33.6%) assayed and analyzed in this data set were well-predicted. Interestingly, we observed substantial overlap in the identities of the metabolites that were well-predicted and anti-predicted in this data set with those predicted similarly in data sets 1 and 2, as well as a general positive correlation between prediction levels across data sets (Fig. 5). One well-predicted metabolite of interest is gamma-aminobutyrate (GABA), which was enriched in the subset of samples from mice 6 weeks after antibiotic treatment. Key contributor analysis indicated that increased synthesis from 4-aminobutanal by an OTU in the genus *Oscillospira* and a *Clostridiales* OTU drove the CMP score variation for this metabolite. Several products of carbohydrate metabolism were also well-predicted, including the sugars stachyose and mannose. Analysis of key contributors revealed that the oligosaccharide stachyose is predicted on the basis of its depletion by glycosidases from diverse *Firmicutes* taxa, including *Ruminococcus* and *Turicibacter*, while mannose is predicted on the basis of increased production via glycan degradation from mannoglycans by several OTUs in the *Clostridiales* order in healthy samples. These shifts reflect the impacts of increased glycan degradation potential in the microbiome of mice from the control cohort compared to those treated with antibiotics. As in the BV data sets, synthesis products found to be more abundant in the more diverse microbiome of the control cohort were most likely to be predictable (48% of 29 such metabolites were well-predicted).

**FIG 5:**
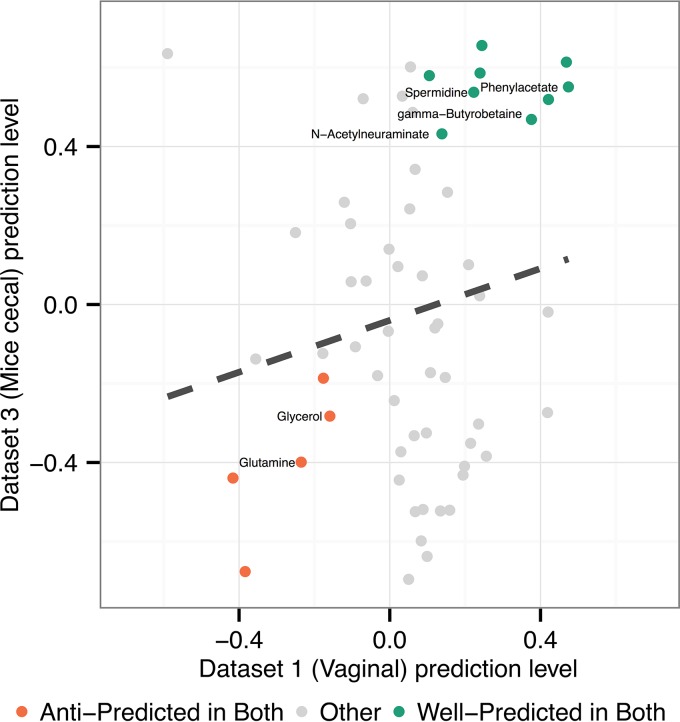
Metabolite predictability is consistent between vaginal and mouse cecal data sets. The plot shows the relationship between the level of predictability for each metabolite (measured as the Spearman correlation between pairwise differences in calculated CMP scores and pairwise differences in measured metabolite abundances) in data set 1 (human vaginal microbiome samples) and data set 3 (mouse gut samples). Colors indicate metabolites that are well-predicted in both data sets or anti-predicted in both data sets. Metabolites that are well-predicted in both data sets are enriched for amino acid catabolites, including phenylacetate, spermidine, and beta-alanine.

In data set 4, only a very low proportion of the metabolites analyzed were well-predicted (6 of 31), which is likely due to a markedly smaller sample size and potentially noisier metabolomic data and identifications. Interestingly, however, four of these six metabolites (chenodeoxycholate, glycochenodeoxycholate, glycocholate, and taurocholate) are primary conjugated or unconjugated bile acids, which form part of a tightly regulated pathway of host-microbial cometabolism with hormonal signaling functions. This enrichment of bile acid-associated products among the well-predicted metabolites (*P* = 0.03; Fisher exact test) highlights the important role of microbiome ecology in microbial metabolism of bile acids in the gut. Specifically, higher levels of bile acid metabolites in irritable bowel disease cases have been noted previously in this data set (41). Our results show that this shift in bile acids is concordant with variation in the abundances of microbial bile salt hydrolase genes.

## DISCUSSION

Above, we have introduced a novel analytical framework that represents an important step toward a principled systematic and mechanistic integrative analysis of microbial community composition and metabolomic data. Our framework goes beyond *ad hoc* correlation-based analysis and aims to assess the correspondence between ecology and metabolic phenotype based on the existing body of knowledge about microbial genomes and metabolic capacities. By evaluating metabolite variation in terms of the functional implications of ecological shifts, we identified a large share of the vaginal metabolome whose variation can be explained by shifts in ecology-based and community-wide enzymatic potential. This high predictability is somewhat surprising, as our framework ignores many factors that could potentially impact this link, including strain variation, gene and protein expression, and metabolic fluxes (14, 43, 44). This finding suggests that ecological dynamics and their impact on community metabolic capacities likely play a major role in mediating broad metabolic differences between microbiomes.

Furthermore, our characterization of key species and gene contributors to calculated CMP scores and, consequently, to the predictability of each metabolite provides evidence that particular BV-associated species have substantial effects on the metabolome. By comprehensively identifying species whose enzymatic capacity and variation across samples are consistent with the observed shift in the abundance of a particular metabolite, we were able to gain deeper insight into the drivers of species-metabolite dynamics in the vaginal microbiota and bacterial vaginosis. Specifically, our analysis of key species contributors identified a subset of BV-associated species (*Eggerthella* sp. 1, *Megasphaera* type 1, and *Mageeibacillus indolicus*) as particularly likely to be important drivers of metabolic variation in this environment. The low contributions of lactobacilli suggest that their importance in the vaginal microbial ecosystem is not described well by current reference knowledge of their role in canonical pathways. Alternatively, this can be attributed to having twice as many women with BV as without BV in our data sets, of which only some women without BV had abundant *L. crispatus*. More generally, we observed that the abundance of metabolic capacities (based on taxonomic composition) is often sufficient to explain measured variability in the abundance of many BV-associated metabolites. This intriguing result suggests that while information about ecological shifts may not necessarily provide a comprehensive understanding of changes in flux in core metabolic reactions, it is often sufficient to account for the accumulation or depletion of many more peripheral metabolites that vary most dramatically between health and dysbiosis.

We also extended this method to analyze data sets from the gut microbiota of mice and humans and identified preliminary mechanistic links in these complex environments. The lower predictability in this context likely reflected the greater complexity of these communities and the plethora of factors, both external and internal to the community, that can potentially affect metabolite abundances. Studying the impact of such factors on various metabolic processes is an important direction for future research. Nevertheless, the overlap observed here in the set of metabolites that are well-predicted across a single organism in culture (*E. coli*), a simple community (the vaginal microbiome), and a complex host-associated community (gut microbiome) may represent shared control points in microbial metabolic networks. This consistency indicates that across multiple environments, the limiting factor for accumulation or depletion of these metabolites is the presence or abundance of microbial enzymatic potential that can directly act on them. This finding further reinforces the credibility of our framework and the shared features of microbial metabolic regulation across all of these settings. In addition, the predictability of the metabolic shifts associated with major ecological perturbations across data sets is consistent with previous metabolic regulation findings that core reactions tend to be regulated by a precise balance between precursor metabolite concentrations and enzyme concentrations, and that intracellular concentrations of core metabolites are generally robust in response to perturbations (45, 46).

One obvious caveat of our framework and of the resulting findings is the inability to distinguish between failure to predict due to missing reference information (e.g., incomplete genome annotation) and failure due to a range of alternative mechanisms regulating metabolite shifts and environmental inputs and outputs. For example, our framework currently does not capture host metabolism, and future work may extend our model to include human gut metabolic processes. Similarly, our model does not consider signaling processes, transcriptional regulation, or bounds on metabolic fluxes. This limitation is further compounded by the use of a broad reaction database, such as KEGG. For example, our model only assigns effects for enzymes catalyzing nonreversible reactions. This approach presumably captures major metabolic fluxes for well-characterized microbes, but the information lost from reversible reactions may hinder our ability to predict metabolites in other pathways. An extended framework could, for example, infer reaction directionality from pathway context or constraint-based modeling, or directly from metabolomic data using a machine learning approach.

Such improvements could also help clarify the interpretation of anti-predicted metabolites, which spanned roughly a third of all predictions across data sets and can be explained by several potential mechanisms. Anti-predicted metabolites whose CMP scores are driven by degradation reactions, especially with downstream well-predicted metabolites, are suggestive of environmentally regulated metabolite changes that cause taxonomic shifts based on nutrient availability, such as the example of glycerol anti-predicted by *L. iners*. Other anti-predicted metabolites may be explained by missing reaction information. For example, putrescine and cadaverine are both anti-predicted based on a high correlation with the abundance of genes coding for enzymes that utilize these metabolites to synthesize further polyamine derivatives (*N*-acetylputrescine and aminopropylcadaverine, respectively). This finding suggests that other enzymes that are currently not incorporated into our predictions (including synthesis reactions that are present but lacking information on reaction directionality) or enzymes from other, unmeasured microbes in these samples are likely important for driving the accumulation of these metabolites. In other cases, anti-prediction may suggest alternative metabolic mechanisms controlling metabolite variation, beyond direct enzymatic regulation.

Finally, the trends revealed by our analysis highlight the tight coordination of various metabolic processes even in the context of complex communities. Our framework evaluated each metabolite independently, but the resulting predictability trends, as well as evidence from other studies (14, 22), show that dramatic shifts in metabolite abundances occur in a strongly coordinated fashion, through a combination of changes in substrate and enzyme concentrations mediated by a variable range of taxa. The analysis framework presented here is an important first step toward deconstructing and interpreting these relationships in mechanistic detail from comprehensive multi-omic data. In turn, this mechanistic understanding will be vital to ultimately enable the rational design of strategies to modify the microbiome and its metabolic phenotype (47, 48).

## MATERIALS AND METHODS

### Assembling and processing data sets.

We obtained several previously published data sets (15, 22, 23, 41, 45) from publicly available databases or through a collaboration, each pairing 16S rRNA gene-based taxonomic data with metabolomic profiles. For vaginal samples, DNA was extracted for 16S rRNA gene analysis from vaginal swabs, and cervicovaginal lavage fluid was collected for metabolomic analysis. Samples from the first data set (data set 1) were analyzed for taxonomic composition using quantitative PCR (qPCR) with primers and probes specific for 14 vaginal bacterial species and for metabolites using global liquid and gas chromatography coupled with mass spectrometry for metabolomics. Samples from the second data set (data set 2) were analyzed by using broad-range 16S rRNA gene PCR coupled with high-throughput 454 sequencing of the 16S rRNA gene (Roche) and targeted metabolomics using LC-MS with multiple-reaction monitoring for 180 compounds, chosen partially based on findings from data set 1. In data set 3, taxonomic composition was assayed using 454 FLX Titanium sequencing of V3-V5 regions of the 16S rRNA gene, and metabolites were measured using global LC-MS and GC-MS metabolomics. Data set 4 paired Roche 454 shotgun sequencing of sample DNA with Fourier transform ion cyclotron resonance mass spectrometry metabolomics. See Table S1 for details about each data set. Metabolite and transcript (microarray) data for the *E. coli* data set were downloaded from the supplementary material of reference 45 and from the NCBI GEO database, profiling *E. coli* grown in culture, treated to cause five different stress-based perturbations, and assayed before and after perturbation. We included only one time point before perturbation and one immediately after for each biological replicate in our analysis. We mapped identified metabolite names to KEGG identifiers (IDs) following the same approach as for data set 2.

We reprocessed 16S rRNA taxonomic sequencing data sets via a standard closed-reference OTU-picking pipeline using QIIME version 1.8.0 (49–53) and rarefied the resulting OTU tables to the number of reads in the lowest-coverage sample. For data set 2, we confirmed that this pipeline produced taxonomic profiles similar to the ones from the *pplacer* method used in the original publication (the Pearson correlation across samples of all genera quantified by both methods was 0.97). We did not normalize the 16S rRNA gene qPCR data of data set 1. A subset of samples in data set 1 also had associated 16S rRNA gene sequencing data, on the basis of which we removed one outlier sample whose sequencing results were dominated by a species not profiled by qPCR. For the *E. coli* gene expression data, we used the KEGG application programming interface (API) to map gene IDs to KEGG orthology groups (KOs) for consistency with the other data sets and used the normalized microarray intensities provided.

Processing of metabolomic data varied depending on the technology used. For data sets 1 and 3, in which metabolomic profiles were produced by Metabolon, Inc., and included detailed metabolite identifications, we filtered out compounds without KEGG identifications and used the raw peak area values. For data set 2 (generated at the Northwest Metabolomics Research Center) and the *E. coli* data set, we used the KEGG API (37) to associate named and measured compounds with KEGG metabolite IDs. For data set 4, which lacked confident library-based metabolite identifications, we used MetaboSearch (54) to perform a mass-based search against the Metlin, MMCD, LipidMaps, and HMDB databases (55–58), with a matching threshold of 1 ppm. The KEGG identifications with the smallest mass difference were assigned as the putative identification, following Tong et al. (24, 33). When multiple putative identifications had the same difference in mass from a peak, preference was given to metabolites in the metabolic network generated based on species abundances, using genomic information as additional support for the presence of that metabolite. When multiple putative KEGG identifications remained, one was randomly assigned to that peak. If multiple peaks mapped to the same KEGG metabolite ID, their abundances were summed. Metabolites with nonzero abundance in <5 samples were discarded from downstream analysis.

### Predicting metagenome content from taxonomic composition.

For data sets 2 and 3, we used PICRUSt (35) to predict metagenome content across samples, based on taxonomic composition, and normalized the resulting predictions using MUSiCC (release 1.0) (36). To predict genome content from the qPCR species abundances in data set 1, we searched IMG for available reference genomes. For 11 of the 14 species profiled, at least one reference genome was available. When multiple reference genomes were available for a given species, we selected either the highest quality genome or a genome from a vaginal isolate (in consultation with the researchers that generated this data set). See Table S2 in the supplemental material for details about the genomes used. We downloaded KEGG orthology (KO) annotations for these genomes from IMG (January 2014) and predicted the metagenome as a product of the reference genome KO annotations and species abundances. For data set 4, orthology group abundances were estimated directly from shotgun sequencing reads using a BLAST-based annotation pipeline (42).

### Metabolic network reconstruction and CMP score calculation.

We adapted the predicted relative metabolomic turnover (PRMT) method developed by Larsen et al. (31) to estimate the metabolic potential of a microbial community based on measurements of gene content. This method does not predict metabolite fluxes or concentrations directly; instead, it synthesizes and integrates information about gene abundances in terms of KEGG orthology groups and a stoichiometric matrix describing the quantitative relationship between genes and metabolites to provide an estimate of the way the community composition may impact each metabolite’s abundance.

To this end, we first created a modified stoichiometric matrix *M* in which each row represents a particular metabolite and each column represents a particular gene (KO), such that each cell *M_ij_* represents the combined relative capacity for enzymatic gene *j* to modify metabolite *i* (see reference 31)*.* To create this matrix, we utilized pathway reaction information and stoichiometric coefficients from KEGG (37). Specifically, for each reaction catalyzed by an enzyme coded by gene *x* that transforms metabolite *A* into metabolite *B* with stoichiometric coefficients *c* and *d*, respectively, we subtract *c* from *M_Ax_* and add *d* to *M_Bx_*. To focus our analysis on the primary transformations catalyzed by each enzyme, we only linked genes to reactions and metabolites that are annotated in KEGG metabolic pathways, using the *reaction_mapformula.lst* file from the KEGG database (2013 version). We then filtered this matrix to only include reactions annotated as occurring in a single direction, ignoring all reversible reactions that cannot contribute to metabolite predictions and all metabolites involved in only reversible reactions. Lastly, we performed two additional modifications: first, following previous studies (59, 60), we excluded “currency” metabolites that are involved in reactions associated with 30 or more genes from the final matrix, and second, following Larsen et al. (31), we normalized each row of *M* such that all negative elements sum to −1 and all positive elements sum to 1.

The resulting matrix accordingly estimates the relative contribution of each gene to the accumulation or depletion of each metabolite. We then multiply this matrix *M* with a matrix *G* that represents the abundance of each gene in each sample to obtain communitywide metabolic potential (CMP) scores, capturing the relative capacity of the metagenome content of each sample to create or deplete each metabolite.

### Comparing CMP scores with metabolomic data.

Notably, since CMP scores represent relative predictions, they can only be interpreted in the context of comparisons between samples (assuming some baseline metabolite profile across samples). Accordingly, to assess how the CMP scores obtained compare to the metabolomic variation measured, we performed a Mantel test for each metabolite, assessing the correlation between pairwise differences (across all pairs of samples) in CMP scores and the corresponding pairwise differences in measured metabolite abundances. We further corrected for multiple hypothesis testing using a local false discovery rate (FDR) approach implemented in the R package *qvalue* (61) and classified metabolites with both a Mantel *P* value and FDR *q* value of less than 0.01 as well-predicted. We evaluated the significance of negative pairwise correlations in a similar manner, classifying metabolites as anti-predicted based on the same significance cutoffs.

### Testing significance with randomly shuffled networks and metabolite labels.

Given the covariance structure of the data set, we also wished to quantify whether our framework identified more well-predicted metabolites than expected by chance. To this end, we repeatedly generated randomized metabolic networks, ran our framework as detailed above using the randomized network to link genes to metabolites, and compared the number of well-predicted metabolites obtained with these randomized networks to the number of well-predicted metabolites obtained with the original network. To preserve the core structural characteristics of the original network, random networks were generated following the edge-shuffling approach outlined in reference 62 (exchanging edges 5,000 times to produce each network).

We also used a permutation-based approach to evaluate whether anti-predicted metabolites are linked by a metabolic reaction to well-predicted metabolites more frequently than expected by chance. To this end, we repeatedly permuted the labels of every metabolite in the network while maintaining a fixed network topology. We counted the number of times an anti-predicted metabolite was connected to a well-predicted metabolite by a synthesizing reaction, a depleting reaction, or a reversible reaction in these permuted networks and compared the resulting distribution with the numbers obtained using the original data.

### Identifying key species and gene contributors.

To quantify the contribution of each species to the calculated CMP score of each metabolite and to identify key species contributors, we examined the Pearson correlation between the CMP scores obtained for a given metabolite across samples using the entire community and the CMP score calculated based on each species by itself (i.e., recalculating the metagenome content and CMP scores based solely on the abundance of each species separately). Species for which this correlation coefficient for a given metabolite was >0.5 were considered key species contributors for that metabolite.

To compare key species contributors between data sets 1 and 2, we identified corresponding species across the two data sets by searching the Greengenes 97% OTU representative sequence set for exact matches with the PCR primers used by Srinivasan et al. (22) to generate data set 1. Notably, this mapping identified OTU 4377809 as *Mageeibacillus indolicus* (previously known as BVAB3), OTU 227000 (mistakenly characterized as *Shuttleworthia*) as BVAB1, and OTU 133178 as *Eggerthella* sp. 1.

To identify key gene contributors to the calculated CMP scores for each metabolite, we examined the Pearson correlation between the CMP scores obtained for a given metabolite across samples using the original stoichiometric matrix and the CMP scores calculated when using a matrix in which the link between the gene in question and the metabolite was deleted (i.e., zeroing the corresponding entry in the matrix). Genes for which this correlation was <0.5 were considered key contributors for that metabolite. We further defined any reaction catalyzed by the enzyme coded by that gene as a key reaction contributor. In addition, if all of the key reaction contributors of a given metabolite produce that metabolite, we classified that metabolite’s CMP scores as driven primarily by synthesis. We similarly classified a metabolite whose key reaction contributors all deplete that metabolite as driven primarily by degradation.

### Data availability.

All data sets analyzed in this paper are from published work, and the relevant sequence data can be found at NCBI under accession numbers SRA051298 (data set 1), SRP056030 (data set 2), SRP033403 (data set 3), and PRJNA46321 (data set 4). The *E. coli* data analyzed are available from the supporting information of the pertaining publication (45) and from NCBI GEO series GSE20305. Taxonomic and metabolomic profiles for each of the data sets analyzed in this work, as well as the code for our framework and analysis, are available at: http://elbo.gs.washington.edu/download.html.
