# Supplementary material for: Metabolic Model-Based Integration of Microbiome Taxonomic and Metabolomic Profiles Elucidates Mechanistic Links between Ecological and Metabolic Variation
Source: mSystems. 2016 Jan 19;1(1):e00013-15. doi: 10.1128/mSystems.00013-15 (PMC4883586; doi:10.1128/mSystems.00013-15)
Supplement: Supplementary file 1 [file sys001160034st9.pdf]

Table S1. Study design and metabolomic data description for all analyzed datasets.

Dataset	Organism and site	Study type	Data used	# samples	# subjects	# metabolites	# KEGG metabolites	# analyzed metabolites	Ref
1	Human, vagina	Case-control (BV)	16S qPCR, metabolomics	67 (42/25/0) *	59 (40/23/0)*	279	197	82	(22)
2	Human, vagina	Case-control (BV)[a]	16S sequencing, targeted metabolomics	70 (40/20/10) *	70 (40/20/10)*	101	96	58	(22)
3	Mouse, gut	Perturbation (antibiotics)	16S sequencing, metabolomics	29		480	297	116	(23)
4	Human, gut (fecal)	Case-control (CD)[b]	Shotgun sequencing, metabolomics	12		1113	136	31	(15, 40)
E. coli	*E. coli* cultures	Perturbation (stress)[c]	Microarray, metabolomics	28		196	87	42	(44)

***** *(BV+/BV-/ intermediate)*
a *Bacterial vaginosis with intermediate samples*
b *Twins discordant and concordant for Crohn's disease*
c *E. coli cultures treated with heat, cold, oxidative stress, glucose-lactose shift*
