# Supplementary material for: Metabolic Model-Based Integration of Microbiome Taxonomic and Metabolomic Profiles Elucidates Mechanistic Links between Ecological and Metabolic Variation
Source: mSystems. 2016 Jan 19;1(1):e00013-15. doi: 10.1128/mSystems.00013-15 (PMC4883586; doi:10.1128/mSystems.00013-15)
Supplement: Supplementary file 2 [file sys001160034s8.docx]

# Supplemental Text

## Predicting variation in metabolites abundances in a simple mono-culture system identifies known mechanisms regulating metabolite variation.

We examined how our metabolic-model based approach performs in a simple mono-culture system with extremely well-characterized metabolic features, as a way to provide a baseline for the boundaries of such an approach. Specifically, we applied our framework to a dataset of transcriptomic (microarray) and metabolomic abundances from *E. coli* culture samples before and after various perturbations (44). As in the more complex systems, we found that a substantial portion of the measured metabolites (26.2%; 11 out of 42) are well-predicted (as defined above) at a 1% false discovery rate (Figure S2A). These well-predicted metabolites include the TCA metabolite succinate, ethanolamine, tyramine, the fatty acid 2-oxobutanoate, GABA (4-aminobutanoate), and 6 amino acids (Figure S2B-G). The key gene contributors for these metabolites coincided with known metabolic regulatory mechanisms. For instance, ethanolamine shifts are predicted by expression changes in the periplasmic glycerophosphodiester phosphodiesterase gene, which is regulated by an operon responsive to phosphodiester availability. Similarly, succinate is predicted by variation in isocitrate lyase expression, which catalyzes the first and main limiting step of the glyoxylate cycle and is repressed during growth on glucose. Tyramine is likewise predicted by expression of the first step of an alternative degradation pathway. Amino acids were generally predicted by a balance between synthesis pathways and depletion by tRNA synthetases. These examples confirm that well-predicted metabolites and the genes that contribute to their predictions are concordant with our knowledge of metabolic network control and perturbation responses in *E. coli*.
